# Evaluating functional C1INH with multiple laboratory methods across Hereditary Angioedema types

**DOI:** 10.3389/fimmu.2025.1654078

**Published:** 2025-08-26

**Authors:** Maine Luellah Demaret Bardou, Rosemeire Navickas Constantino-Silva, Maria Luiza Oliva Alonso, Ana Júlia Ribeiro Teixeira, Pedro Francisco Giavina-Bianchi, Eli Mansour, João Bosco Pesquero, Solange Oliveira Rodrigues Valle, Anete Sevciovic Grumach

**Affiliations:** ^1^ Clinical Immunology, Centro Universitario Faculdade de Medicina ABC, Santo Andre, SP, Brazil; ^2^ Department of Clinical Medicine, Immunology Service, Hospital Universitario Clementino Fraga Filho, Federal University of Rio de Janeiro, Rio de Janeiro, Brazil; ^3^ Division of Allergy and Clinical Immunology, University of São Paulo, São Paulo, SP, Brazil; ^4^ Allergy and Immunology, Department of Internal Medicine, School of Medical Sciences, University of Campinas, Campinas, Brazil; ^5^ Department of Biophysics, Federal University of São Paulo, São Paulo, SP, Brazil

**Keywords:** hereditary angioedema, C1 inhibitor, factor XII, complement C4, biomarker, diagnosis

## Abstract

**Introduction:**

Hereditary Angioedema (HAE) is a rare genetic disease characterized by recurrent episodes of edema and classified into HAE with C1 inhibitor deficiency (HAE-C1INH types 1 and 2) and HAE with normal C1INH (HAE-nC1INH). This study evaluates the function of C1 inhibitor (fC1INH) in patients with suspected HAE using several laboratory methods: dried blood spot (DBS), chromogenic assay, and ELISA with FXIIa and PKa (plasma kallikrein). The comparative approach aims to improve early detection and understanding of C1INH dysfunction in all HAE subtypes to reflect real-world diagnostic scenarios.

**Methods:**

We assessed the diagnostic performance of four fC1INH assays in a cohort of 148 HAE patients: 84 with HAE-C1INH (72 type 1 and 12 type 2) and 64 with HAE-nC1INH (53 HAE-FXII and 11 HAE-UNK). The gold-standard chromogenic assay and the two substrate-specific ELISAs (PKa and FXIIa) were compared to a novel DBS-based LC-MS/MS assay using endogenous C1s activity. For all fC1INH assays, values >50% were considered within the normal range.

**Results:**

In HAE-C1INH, the DBS assay showed the highest specificity (type 1: 98.6%, type 2: 100%) and 100% sensitivity for both subtypes. ELISA-FXIIa also performed well (specificity: 97.2% and 91.7%). In contrast, ELISA-PKa and the chromogenic assay showed reduced specificity in type 2 (25% and 66.7%, respectively). Among patients with HAE-FXII, fC1INH levels were reduced by 36.5% by ELISA-FXIIa (19/52), 19.1% by DBS (9/47), and 3.8% by ELISA-PKa (2/52), and no alterations were detected by the chromogenic assay. Some of the changes seen in other tests may be partly related to pregnancy in a few patients. In the HAE-UNK group, all 11 patients had fC1INH >50% in all methods.

**Conclusion:**

DBS-based LC-MS/MS and ELISA-FXIIa offer promising accuracy and broader applicability for early diagnosis of HAE types 1 and 2. The use of novel substrates and the inclusion of a clinically realistic cohort may enhance the translational relevance of these findings.

## 1 Introduction

Hereditary Angioedema (HAE) is a rare, potentially life-threatening genetic disorder characterized by recurrent, unpredictable episodes of subcutaneous and submucosal edema. The most commonly affected sites include the face, extremities, gastrointestinal tract, genitalia, and upper airways. Misdiagnosis or delayed recognition may result in unnecessary surgical interventions, avoidable morbidity, and, in some cases, airway compromise due to laryngeal edema (1–3).

HAE is classified based on the presence or absence of C1 inhibitor (C1INH) deficiency. HAE-C1INH results from either a quantitative reduction (type 1) or a dysfunctional protein with normal or elevated levels (type 2) (2). In contrast, HAE with normal C1INH (HAE-nC1INH) includes patients with normal antigenic and functional C1INH levels (fC1INH), and in a subset of these cases, pathogenic variants have been identified in genes already known to be associated with the disease.

C1INH is a serine protease inhibitor that regulates the complement, coagulation, fibrinolytic, kinin-kallikrein, and contact systems by inhibiting proteases such as plasma kallikrein (PKa), factor XI (FXI), and factor XII (FXII) (5, 6). C1INH deficiency leads to excessive bradykinin production through uncontrolled PKa-mediated cleavage of high-molecular-weight kininogen (HK), resulting in increased vascular permeability (7, 8).

More than 800 variants in the *SERPING1* gene have been identified in patients with C1INH deficiency (HAE-C1INH) (https://databases.lovd.nl/shared/variants/SERPING1) (9, 10). It is the most prevalent HAE type, with an estimated frequency from 1:50,000 to 1:100,000 (11). HAE-C1INH-Type1 accounts for approximately 85% of cases and is characterized by reduced antigenic and functional levels of C1INH. In HAE-C1INH-Type2, C1INH levels are normal or elevated, but function is impaired. In both subtypes, fC1INH levels are typically <50%, making it a reliable diagnostic biomarker (2). Complement component C4 is usually decreased; however, its diagnostic value is limited due to suboptimal sensitivity and specificity (12–14). While *SERPING1* sequencing may be informative, particularly in early or prenatal contexts, C1INH antigen and function assays are cost-effective and remain first-line diagnostic tools (3, 15, 16).

Diagnosis of HAE-nC1INH relies on molecular testing. The most extensively studied subtype HAE-FXII is caused by variants in the *F12* gene, which increase susceptibility to cleavage by plasma kallikrein and promote excessive bradykinin production (4, 17). Several other rare genetic variants have been identified, including mutations in plasminogen (HAE-PLG) (18), angiopoietin 1 (HAE-ANGPT1) (19), kininogen1 (HAE-KNG1) (20), myoferlin (HAE-MYOF) (21), heparan sulfate 3-O-sulfotransferase 6 (HAE-HS3ST6) (22), carboxypeptidase N (HAE-CPN1) (23), and disabled homologous interacting protein 2 (HAE-DAB2IP) (24). Patients with no identifiable mutations are termed HAE-UNK and typically present with a family history, lack of response to antihistamines or omalizumab, and positive response to HAE-specific treatments (3, 4, 25).

All suspected cases of HAE should be assessed for C1INH antigen, function, and C4 levels (3, 16, 26). However, logistical barriers persist. Complement proteins are thermolabile and require prompt aliquoting and storage at −80°C for accurate results (2, 27, 28). Access to specialized laboratories remains limited, and shipping samples on dry ice incurs high costs (29). Recent innovations include dried blood spot (DBS) sampling coupled with liquid chromatography-tandem mass spectrometry (LC-MS/MS), which has shown high sensitivity and practicality for remote testing (30–33). In this study, we aimed to evaluate different subtypes of HAE using diverse methodologies for assessing fC1INH, with a focus on comparing the sensitivity and specificity of these diagnostic approaches.

## 2 Methods

### 2.1 Study design and setting

This multicenter, cross-sectional study was coordinated by the Centro Universitário FMABC (CEUFMABC) in Santo André, Brazil, an internationally accredited ACARE (Angioedema Center of Reference and Excellence). To ensure a robust and representative cohort, patient recruitment and sample collection were conducted in collaboration with reference centers from the Brazilian Group for the Study of Hereditary Angioedema (GEBRAEH), including the University of Campinas, the Federal University of Rio de Janeiro (Hospital Clementino Fraga Filho), and the University of São Paulo. Standardized protocols were implemented across all participating centers, covering sample collection timing, tube type, centrifugation parameters, storage conditions, and transportation logistics. All samples were centrally processed at CEUFMABC.

### 2.2 Study participants and diagnostic workflow

This study was approved by the Brazilian National Research Ethics Commission (CAAE: 41812720010010082), and written informed consent was obtained from all participants and/or their legal guardians.

We enrolled individuals aged over 1 year who met at least one of the following criteria: 1) a confirmed diagnosis of HAE, 2) presence of suggestive symptoms without a definitive diagnosis, or 3) being an asymptomatic first-degree relative of an index case. Diagnostic classification was based on clinical history, family pedigree, and biochemical evaluation, including C1 inhibitor level (C1INHq), fC1INH, and complement C4.

Exclusion criteria comprised comorbidities known to interfere with complement activity—such as hepatic or renal disease, autoimmune disorders, chronic infections, or hematologic malignancies. Additionally, individuals ultimately diagnosed with mast cell-mediated angioedema during follow-up were excluded from the study.

In patients with suspected HAE and normal C1INH levels and function (HAE-nC1INH), genetic testing with Sanger sequencing of exon 9 of the FXII gene was performed on an ABI 3500 Genetic Analyzer (Applied Biosystems) at the JB Pesquero Molecular Genetics Laboratory, a facility specialized in genomic diagnostics for HAE. When no variant was identified, whole-exome sequencing was conducted to investigate additional known pathogenic variants.

### 2.3 Sample collection and processing

Peripheral blood was collected into sodium citrate, EDTA, and serum tubes. Plasma and serum were separated by centrifugation at 1,207× ***g*** for 10 min at 4°C and stored at −80°C. For DBS analysis, 50 µL of EDTA-anticoagulated whole blood was applied to filter paper, dried at room temperature for ≥3 h, and stored at −20°C until shipment to Revvity Omics (USA) for LC-MS/MS analysis.

### 2.4 Complement quantification and functional C1INH assays

#### 2.4.1 Antigenic quantification of C1INH and C4

C1INHq and C4 were determined by radial immunodiffusion (Binding Site, Birmingham, United Kingdom). Reference values were 19.5–34.5 mg/dL for C1INHq and 20–40 mg/dL for C4.

#### 2.4.2 Functional assay: chromogenic method

Functional C1INH activity was assessed in citrated plasma using the Technochrom^®^ C1INH kit (Technoclone, Vienna, Austria), which measures the ability of C1INH to inhibit cleavage of the synthetic substrate Z-Lys-SBzl·HCl by C1s. The reaction releases free thiol, which reacts with 5,5′-dithiobis-(2-nitrobenzoic acid) (DTNB, Ellman’s reagent), forming a yellow compound quantified spectrophotometrically at 412 nm. Lower absorbance indicates higher C1INH activity. Values above 50% were considered within the normal range.

#### 2.4.3 Functional assay: DBS LC-MS/MS

This method was developed and validated by Revvity Omics (CLIA-certified for high-complexity testing) to quantify cbz-Lys, the cleavage product generated by free C1s. C1INH extracted from DBS cards was incubated with an excess of C1s, and the resulting cleavage products were quantified by LC-MS/MS. Although the manufacturer’s reference value is >62.8%, a cutoff of >50% was adopted based on previously published studies (3, 16).

#### 2.4.4 Functional assay: ELISA with FXIIa and PKa

Two in-house ELISAs were performed as previously described by Joseph et al. (34) to assess functional C1INH activity using biotinylated activated factor XII (FXIIa) and PKa as substrates. Nunc Maxisorp 96-well plates were coated with streptavidin (5 µg/mL) in carbonate/bicarbonate buffer (pH 9.6) and incubated overnight at 4°C. After blocking with PBS containing 1.5% BSA for 2 h at room temperature and washing with PBS-Tween, 50 µL of binding buffer, 25 µL of C1INH standards (R&D Systems, Minneapolis, MN, United States; 5, 2.5, 1.25, 0.63, 0.31, 0.16, and 0 µg/mL), and diluted plasma samples (1:100) were added, followed by 25 µL of biotinylated FXIIa or PKa (Enzyme Research, South Bend, Indiana, United States). The plate was incubated at 37°C for 1 h. After additional washes, HRP-conjugated anti-C1INH antibody (1:10,000; Thermo Fisher, Waltham, MA, United States) was added and incubated for 1 h. The reaction was developed with chromogenic substrate and stopped after 5–10 min. Absorbance was read at 450 nm. Values above 50% were considered within the normal range. General-use reagents (PBS, BSA, buffers, and streptavidin) were purchased from Sigma (Burlington, MA, United States).

### 2.5 Cohort description and diagnostic grouping

A total of 187 biological samples were collected from individuals, including patients with a confirmed diagnosis of HAE, symptomatic and asymptomatic relatives of HAE patients, and individuals with recurrent angioedema of unknown etiology. Twenty-eight participants were excluded from the final analysis due to either negative diagnostic results among relatives (***n*** = 15) or the inability to obtain confirmatory samples (***n*** = 13). In addition, 11 samples from patients initially suspected of having HAE were excluded following diagnostic reclassification as mast cell-mediated angioedema, based on established clinical and laboratory criteria.

The final study cohort of 148 included patients with HAE-C1INH (type 1, ***n*** = 72; type 2, ***n*** = 12) and HAE-nC1INH, comprising HAE-FXII (***n*** = 53) and HAE-UNK (***n*** = 11). Regarding control samples, the number varied according to the method employed: DBS (***n*** = 27, including 15 from relatives with negative results), chromogenic assay (***n*** = 14, all from relatives with negative results), PKa (***n*** = 64), and FXIIa (***n*** = 61) (**Supplementary Table S1**).

Of the 72 individuals with HAE-C1INH-Type1, 51 were female patients (70.8%), with a mean age of 39.8 years (range: 3–79) (**Supplementary Table S2**). Among the 12 patients with HAE-C1INH-Type2, 7 were female subjects (58.3%), with a mean age of 35.5 years (range: 11–56); one woman was experiencing an AE attack at the time of sample collection (**Supplementary Table S3**).

An additional 64 patients were diagnosed with HAE-nC1INH, including 53 with a pathogenic variant in the *F12* gene (HAE-FXII) and 11 without an identifiable mutation (HAE-UNK). Sixteen newly confirmed diagnoses were made within the HAE-FXII group. The majority of HAE-FXII patients were women (37 out of 53; 69.8%), with a mean age of 40 years (range: 11–56), of whom 3 were pregnant at the time of sample collection (**Supplementary Table S4**). All 11 patients in the HAE-UNK group were women (100%), with a mean age of 39.9 years (range: 24–68). These patients had a positive family history of angioedema, comprising three distinct families: seven individuals from family 57, two from family 58, and two from family 59. All responded to HAE-specific treatments but not to therapies targeting mast cell-mediated angioedema. Whole-exome sequencing revealed no known pathogenic variants in this group, except for a variant of uncertain significance (VUS) in the ANGPT1 gene, identified in a mother and daughter from a family with multiple affected members for whom further genetic analysis was not possible (**Supplementary Table S5**).

## 3 Results

**Figure 1:**
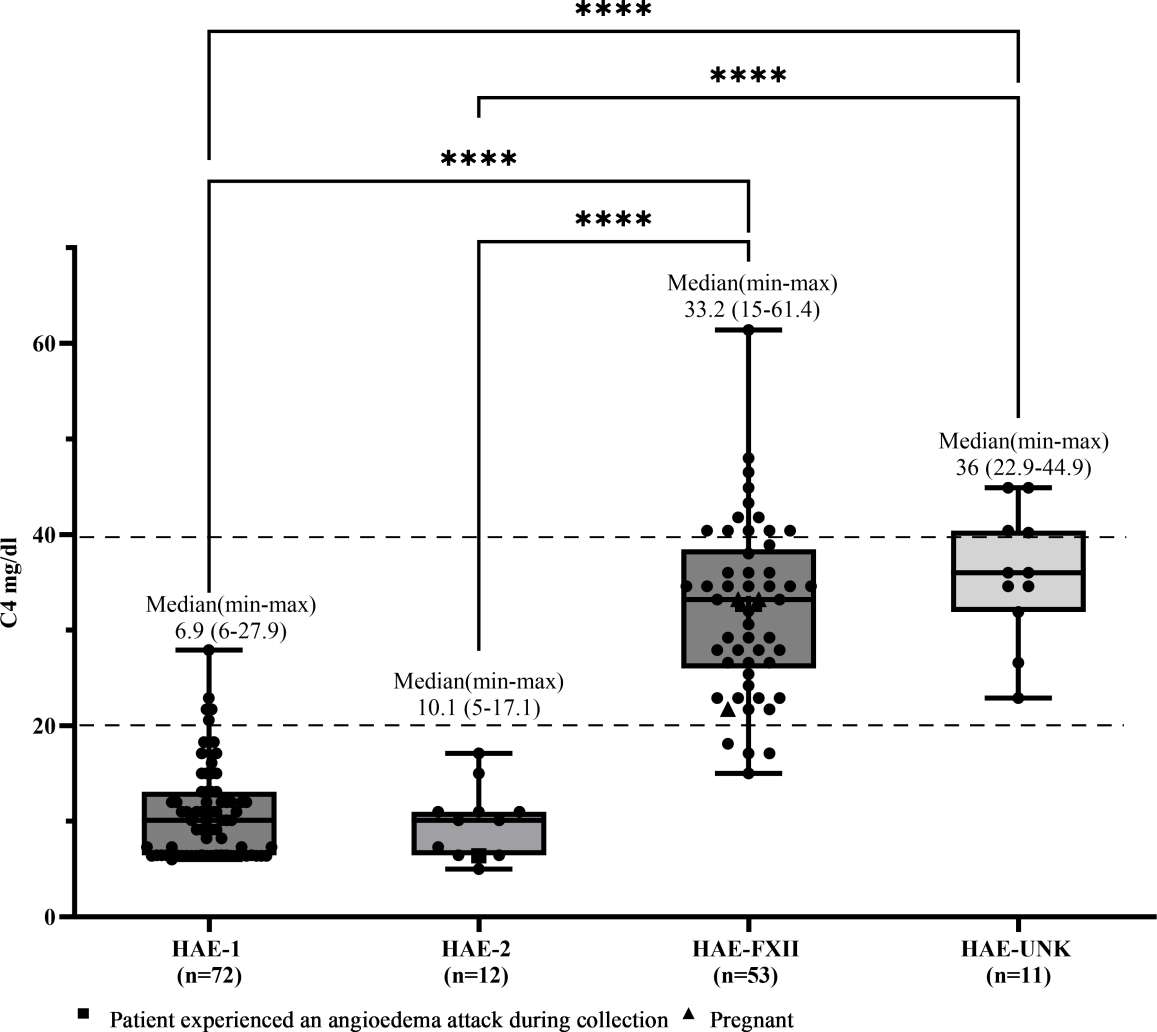
C4 levels according to types of hereditary angioedema. Box-and-whisker plots represent serum C4 concentrations (mg/dL) in patients with hereditary angioedema (HAE) due to C1INH deficiency type 1 (HAE-1) and HAE due to C1INH deficiency type 2 (HAE-2), as well as HAE with normal C1 inhibitor levels, subdivided into HAE with factor XII mutation (HAE-FXII) and HAE of unknown mutation (HAE-UNK). The normal reference interval for serum C4 is indicated by dashed lines. Boxes represent the interquartile range (IQR); the horizontal line within each box indicates the median, and whiskers extend from the minimum to the maximum values. Individual data points are overlaid. Statistical analysis was performed using the Kruskal-Wallis test followed by Dunn's multiple comparisons test. (****p <0.0001).

**Figure 2:**
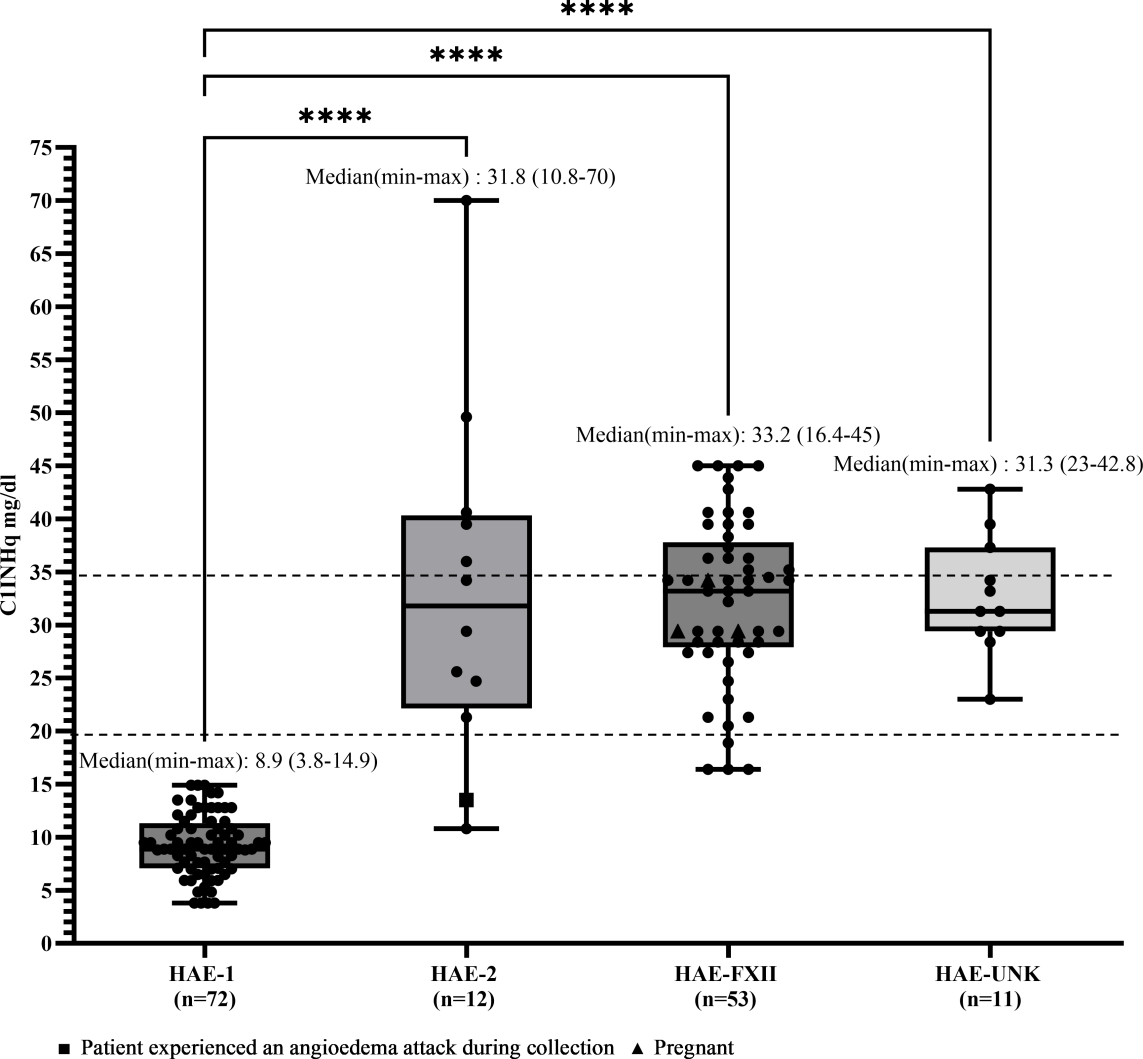
C1INHq levels according to HAE diagnosis. Box-and-whisker plots represent C1 inhibitor level concentrations (CIINIIq, mg/dL) in patients with hereditary angioedema (HAE) due to CIINH deficiency type 1 (HAE-1) and HAE due to C1INH deficiency type 2 (HAE-2), as well as HAE with normal C1 inhibitor levels, subdivided into HAE with factor XII mutation (HAE-FXII) and HAE of unknown mutation (HAE-UNK). The normal reference interval for CIINHq levels is indicated by dashed lines. Boxes represent the interquartile range (IQR); the horizontal line within each box indicates the median, and whiskers extend from the minimum to the maximum values. Individual data points are overlaid. Statistical analysis was performed using the Kruskal-Wallis test followed by Dunn's multiple comparisons test. (****p<0.0001).

Median C4 and C1INHq levels were markedly reduced in patients with HAE-C1INH-Type1, with values of 10.1 mg/dL (range: 6.0–27.9) and 8.88 mg/dL (range: 3.8–14.9), respectively (**Figures 1**, **2**). Reference values were 19.5–34.5 mg/dL for C1INHq and 20–40 mg/dL for C4. Notably, five individuals (unrelated adults aged 28 to 72 years) had C4 values within the elevated range (**Supplementary Table S1**). Patients with HAE-C1INH-Type2 showed similarly low C4 levels (median: 10.1 mg/dL; range: 5.0–17.1), while C1INHq levels were elevated (median: 31.8 mg/dL; range: 10.8–70.0), consistent with the known biochemical profile of this subtype. One patient experiencing an AE attack at the time of sample collection had reduced C1INHq levels. Additionally, one child in this group also had reduced C1INHq levels (**Figures 1**, **2**).

In patients with HAE-FXII, both C4 and C1INHq levels were within the normal range (median for both: 33.2 mg/dL), although one young adult had a decreased C4 level of 15 mg/dL. In the HAE-UNK group, median C4 and C1INHq levels were 36.0 mg/dL (range: 22.9–44.9) and 31.3 mg/dL (range: 23.0–42.8), respectively (**Figures 1**, **2**).

**Figure 3:**
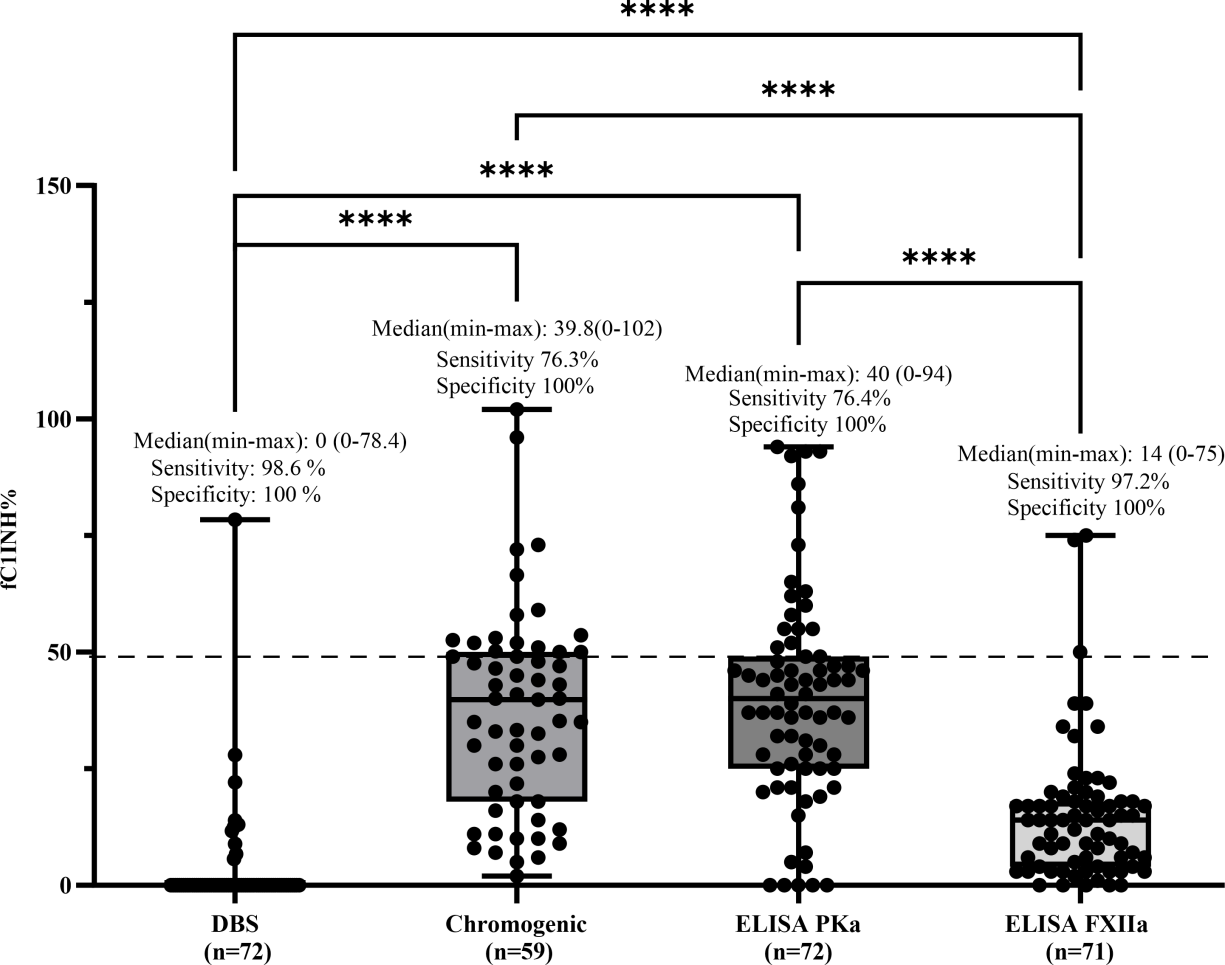
Comparison of fC1INH % values by different methods in HAE-C1INH-Type1. Box-and-whisker plots represent functional C1 inhibitor (fC1INH%) levels in patients with hereditary angioedema due to C1INH deficiency type 1 (HAE-C1INH-Type1), assessed using four different methods: dried blood spot (DBS), chromogenic assay, and ELISAs employing kallikrein (PKa) or activated factor XII (FXIIa) as substrates. The dashed line indicates the lower reference limit for normal fC1INH activity (50%). Boxes represent the interquartile range (IQR); the horizontal line within each box indicates the median, and whiskers extend from minimum to maximum values. Individual data points are overlaid. Statistical analysis was performed using the Kruskal-Wallis test followed by Dunn's multiple comparisons test. (****p < 0.0001). Sensitivity was calculated by identifying true positive results defined as fC1INH activity below 50% in patients with confirmed HAE-1. Specificity was assessed using samples from individuals without C1INH deficiency, where true negatives were defined as fC1INH activity ≥50%.

Sensitivity was calculated as the proportion of true positive (TP) results among patients with HAE-C1INH type 1, using a cutoff of 50% for fC1INH levels. As all patients with HAE-C1INH type 1 are expected to have fC1INH activity below 50%, values ≤50% were considered positive (i.e., abnormal). The number of true positives (TP) for each method was 71/72 for the DBS assay, 45/59 for the chromogenic assay, 55/72 for the ELISA PKa method, and 69/71 for the ELISA FXIIa method. Sensitivity was expressed as TP/(TP + FN), with false negatives (FN) defined as patients with a confirmed diagnosis of HAE-C1INH type 1 who unexpectedly showed fC1INH values above 50% and were therefore misclassified as normal. Among the functional assays, the DBS method showed the highest sensitivity for detecting fC1INH deficiency in HAE-C1INH type 1 patients (71/72; 98.6%), followed by ELISA-FXIIa (69/71; 97.2%), the chromogenic assay (45/59; 76.3%), and ELISA-PKa (55/72; 76.4%). Median fC1INH values (range) were as follows: DBS, 0% (0–78.4); chromogenic, 39.8% (2–102); ELISA-PKa, 40% (0–94); and ELISA-FXIIa, 14% (0–75) (**Figure 3**).

**Figure 4:**
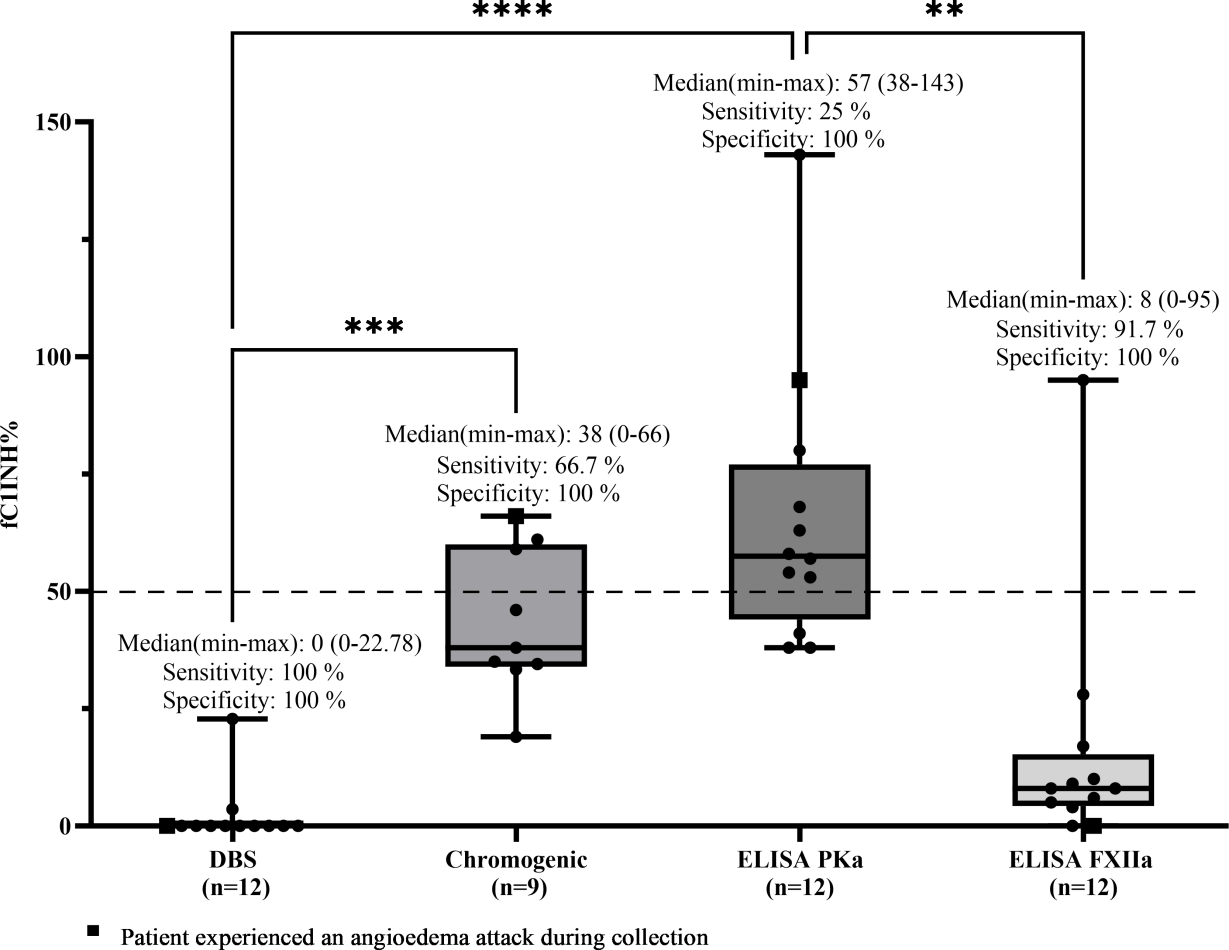
Comparison of fC1INH % values by different methods in HAE-C1INH-Type2. Box-and-whisker plots represent functional C1 inhibitor (fC1INH%) levels in patients with hereditary angioedema due to C1INH deficiency type 2 (HAE-C1INH-Type2), assessed using four different methods: dried blood spot (DBS), chromogenic assay, and ELISAs employing kallikrein (PKa) or activated factor XII (FXIIa) as substrates. The dashed line indicates the lower reference limit for normal fC1INH activity (50%). Boxes represent the interquartile range (IQR); the horizontal line within each box indicates the median, and whiskers extend from minimum to maximum values. Individual data points are overlaid. Statistical analysis was performed using the Kruskal-Wallis test followed by Dunn's multiple comparisons test (**p < 0.01; ***p < 0.001; ****p < 0.0001); Sensitivity was calculated by identifying true positive results-defined as fC1INH activity below 50% in patients with confirmed HAE-1. Specificity was assessed using samples from individuals without C1INH deficiency, where true negatives were defined as fC1INH activity ≥50%.

For patients with HAE-C1INH type 2, sensitivity was calculated using the same rationale, since these patients also present with functional deficiency of C1 inhibitor, despite having normal or elevated antigenic levels. Therefore, all values ≤50% were considered true positives. Any result >50% was interpreted as a false negative, representing an inappropriately normal result in a patient with a confirmed diagnosis. Based on these criteria, the DBS method correctly identified all 12 patients with values ≤50%, resulting in a sensitivity of 100%. ELISA-FXIIa detected 11 out of 12 cases (sensitivity of 91.7%), the chromogenic method identified 6 out of 9 patients (66.7%), and ELISA-PKa detected only 3 out of 12 (25%). Median values (range) in this group were as follows: DBS, 0% (0–22.78); chromogenic, 38% (0–66); ELISA-PKa, 57% (38–143); and ELISA-FXIIa, 8% (0–95) (**Figure 4**).

**Figure 5:**
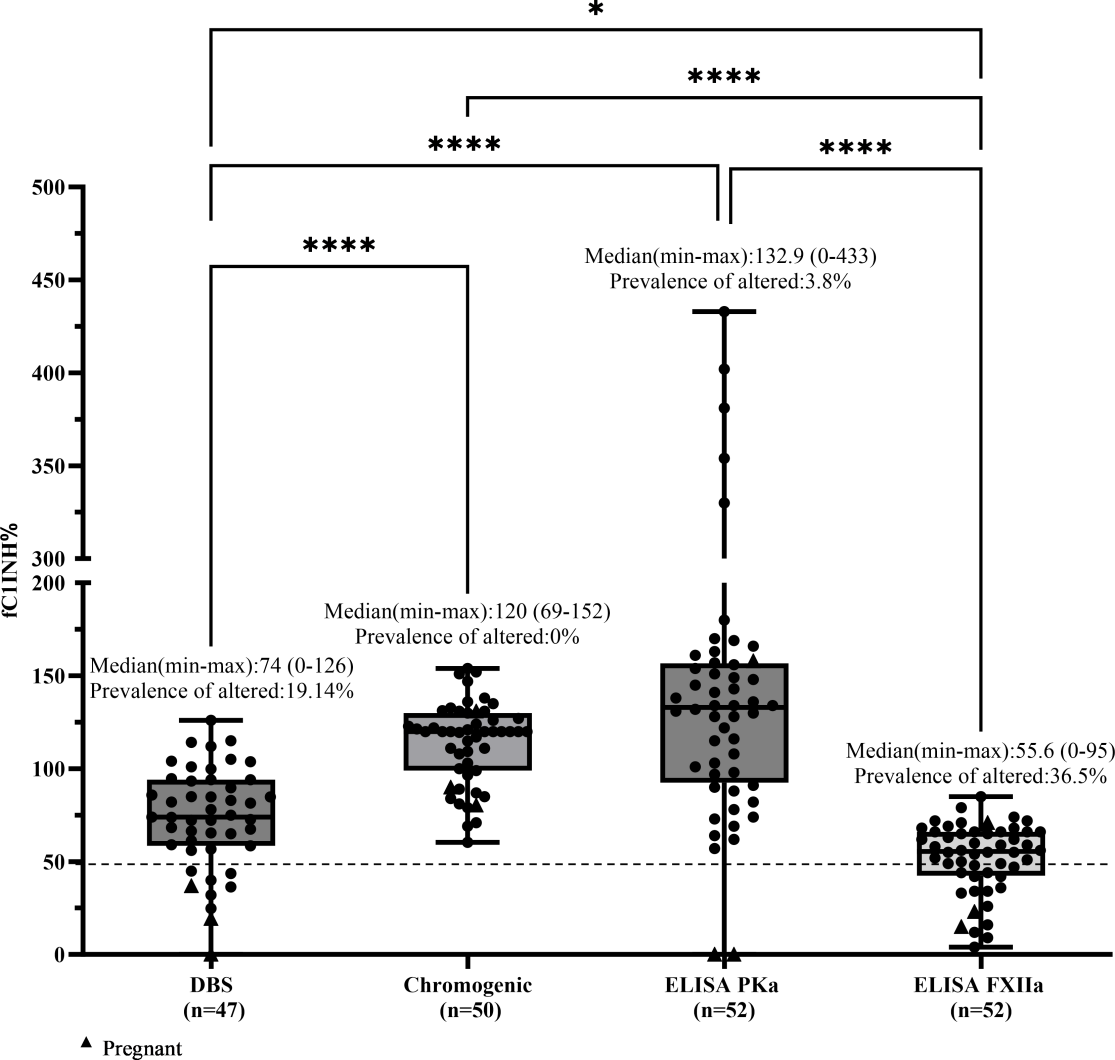
Comparison of fC1INH % values by different methods in HAE-FXII. Box-and-whisker plots depict functional C1 inhibitor (fC1INH%) levels in patients with HAE with normal CIINH due to a Factor XII variant (HAE-FXII), assessed using four different methods: dried blood spot (DBS), chromogenic assay, and ELISAs employing kallikrein (PKa) or activated factor XII (FXIIa) as substrates. The dashed line represents the lower reference limit for normal fC1INH activity (50%). Boxes indicate the interquartile range (IQR); the horizontal line within each box shows the median, and whiskers extend from the minimum to maximum values. Individual data points are overlaid. Statistical analysis was performed using the Kruskal-Wallis test followed by Dunn's multiple comparisons test (*p < 0.05; ****p < 0.0001).

In contrast, patients with HAE-FXII and HAE of unknown cause (HAE-UNK) are expected to have normal fC1INH function. For these groups, values below 50% were not expected and were therefore interpreted as altered or falsely abnormal results. In these cases, the prevalence of altered results was calculated, defined as the proportion of individuals with fC1INH values ≤50%. In the HAE-FXII group (*n* = 47), the DBS method showed altered values in 9 patients (19.1%, including all 3 pregnant women in the study), the chromogenic method in none (0%), ELISA-PKa in 2 out of 52 patients (3.8%, both pregnant), and ELISA-FXIIa in 19 out of 52 patients (36.5%, including 2 pregnant women). Median fC1INH values (range) were as follows: DBS, 74% (0–126); chromogenic, 120% (69–152); ELISA-PKa, 132.9% (0–433); and ELISA-FXIIa, 55.6% (0–95) (**Figure 5**).

**Figure 6:**
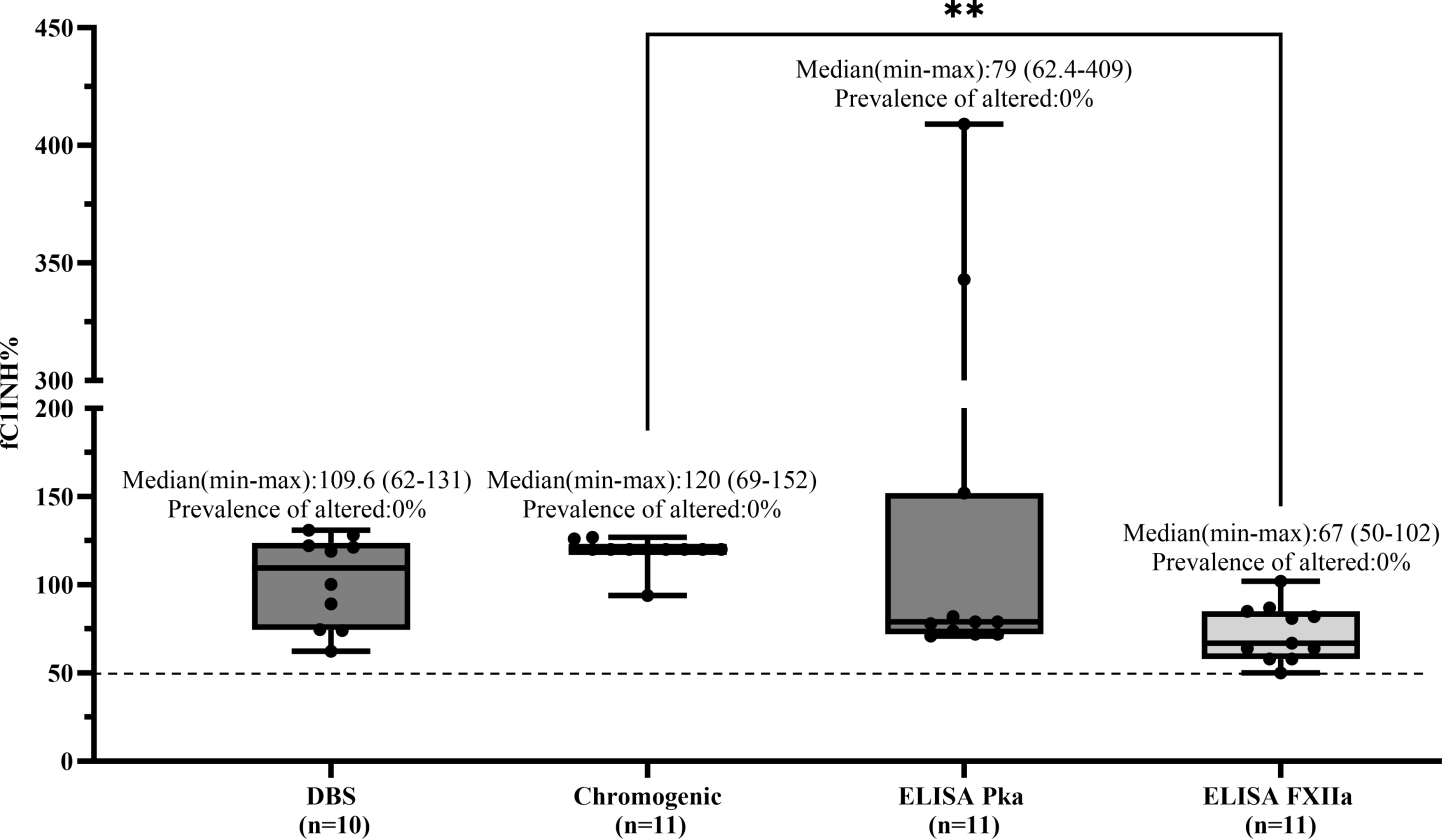
Comparison of fC1INH % values by different methods in HAE-UNK. Box-and-whisker plots depict functional C1 inhibitor (fC1INH%) levels in patients with HAE with normal C1INH with unknown mutation (HAE-UNK), assessed using four different methods: dried blood spot (DBS), chromogenic assay, and ELISAs employing kallikrein (PKa) or activated factor XII (FXIIa) as substrates. The dashed line represents the lower reference limit for normal fC1INH activity (50%). Boxes indicate the interquartile range (IQR); the horizontal line within each box shows the median, and whiskers extend from the minimum to maximum values. Individual data points are overlaid. Statistical analysis was performed using the Kruskal-Wallis test followed by Dunn's multiple comparisons test (**p < 0.01).

In the HAE-UNK group (*n* = 11), all patients showed fC1INH levels above 50% across all methods, resulting in a prevalence of altered values of 0% for all assays. Median values (range) were as follows: DBS, 109.6% (62–131); chromogenic, 120% (69–152); ELISA-PKa, 79% (62.4–409); and ELISA-FXIIa, 67% (50–102) (**Figure 6**).

Specificity was defined as the proportion of true negative results among healthy controls. All control individuals had fC1INH levels >50% across all methods evaluated and were therefore classified as true negatives (TN). As no false positives (FP) were observed, specificity was calculated as TN/(TN + FP), resulting in 100% for all assays tested.

## 4 Discussion

HAE remains underrecognized globally, especially in settings with limited access to specialized laboratory testing (29, 35). Although clinical criteria are well established, biochemical confirmation remains critical for accurate subtype differentiation and therapeutic decision-making. Standard diagnostics rely on the evaluation of C4 and C1INH antigenic and functional C1INH levels. However, these markers are variably affected by pre-analytical factors such as sample handling and storage, which may reduce the reliability of results (27, 28, 36, 37).

This study presents novel comparative methods of evaluation of fC1INH performed in a population of patients that closely mirrors real-world clinical scenarios. Unlike many previous studies that stratify patients only after the complete diagnostic workup, this work reflects the practical reality: all patients with suspected HAE initially undergo functional C1INH testing, regardless of their final classification (HAE-C1INH or HAE-nC1INH). This approach enhances the applicability and relevance of the findings to early diagnostic pathways. A key methodological innovation of this study lies in the head-to-head comparison of multiple fC1INH assays, DBS, chromogenic assay, and ELISA, using distinct substrates. While both DBS and chromogenic assays utilized C1s, in line with standard diagnostic protocols, the in-house ELISA developed specifically for this research employed novel substrates: activated factor XII (FXIIa) and plasma kallikrein (PKa) (34). These components target the kinin-kallikrein system (KKS), offering a unique perspective on C1INH functionality. This innovative use of FXIIa and PKa not only broadens the understanding of KKS regulation but also suggests the potential for greater sensitivity in detecting functional abnormalities, particularly in diagnostically challenging cases.

In HAE-C1INH-Type1, patients are expected to have uniformly reduced levels of C4, C1INHq, and fC1INH. Our findings largely confirm this biochemical pattern: 72 patients classified as type 1 exhibited low median values across all markers, and the majority had fC1INH <50% by DBS, ELISA-FXIIa, chromogenic, and ELISA-PKa. However, exceptions were noted: five type 1 patients (one child and four adults) had C4 within or above the reference range, underscoring the limitations of relying on a single marker (3, 12).

In HAE-C1INH-Type2, the classic profile includes reduced C4, normal or elevated C1INHq, and diminished function. Our data support this: the median C1INHq was elevated with a normal range extending up to 70 mg/dL, while C4 remained low. Functionally, only DBS (100%) and ELISA-FXIIa (91.7%) reliably detected reduced fC1INH, while the chromogenic assay (66.7%) and ELISA-PKa (25%) showed low or very low sensitivity.

Notably, while the chromogenic assay remains the conventional reference, our data confirm its limited sensitivity for HAE-C1INH-Type2 and variable performance in type 1. This may reflect assay vulnerability to pre-analytical influences, as described in the literature (37–39). Our results suggest that reliance on this assay alone may miss a substantial proportion of affected individuals, particularly in the presence of borderline or fluctuating levels.

In contrast, the DBS LC-MS/MS method and ELISA-FXIIa assay demonstrated superior diagnostic performance across both subtypes. DBS detected nearly all functional deficiencies in types 1 and 2, offering advantages of simplified logistics and greater reproducibility, even in decentralized settings. While the DBS approach offers logistical benefits such as minimal sample volume, ambient stability, and remote usability, its implementation is still constrained by the need for LC-MS/MS infrastructure, a high-complexity technology still unavailable in many laboratories and countries, particularly in low-resource settings (30–33). ELISA-FXIIa also showed high accuracy, though its in-house nature and lack of commercial availability remain barriers to broad implementation. However, these two assays were less specific in the HAE-FXII group, where fC1INH levels are expected to be normal. Apparent reductions in fC1INH were observed in 19.1% of cases by DBS and in 36.5% by ELISA-FXIIa, particularly among pregnant women, likely reflecting expected physiological changes during pregnancy, such as hormonal modulation or increased C1INH consumption. These findings underscore the high sensitivity of the assays in detecting altered function; however, no clear explanation has been established for other isolated reductions observed in non-pregnant individuals (17, 40).

In patients with HAE-UNK, all assays resulted in normal fC1INH levels, and the group showed biochemical profiles consistent with preserved complement function, reinforcing the need for genetic exploration and assessment of treatment response for diagnosis (3, 4).

Finally, our findings reinforce the limited utility of C4 as a standalone screening marker. Although historically used in the initial evaluation of HAE, its sensitivity and specificity are suboptimal. Notably, several genetically confirmed cases exhibited normal C4 levels, highlighting that a normal C4 result does not exclude the diagnosis of HAE. Therefore, C4 measurement should be repeated during symptomatic episodes to improve diagnostic accuracy (12).

The strengths of this study include its multicenter design, a large and well-characterized cohort with confirmed genetic diagnoses, standardized sample processing, and comparative analysis of four distinct functional assays. The inclusion of controls provided an internal benchmark for specificity and variability.

In summary, this work supports the integration of functional assays—especially DBS and ELISA-FXIIa—into diagnostic workflows and cautions against overreliance on C4 or chromogenic testing alone. A combination of clinical evaluation, comprehensive complement testing, and genetic confirmation remains essential for accurate diagnosis and optimal care.

## 5 Conclusion

This study demonstrates that both the DBS LC-MS/MS method and the in-house ELISA-FXIIa assay offer high diagnostic accuracy for HAE-C1INH, where conventional assays such as the chromogenic method may lack sensitivity. DBS stands out for its practical advantages, such as simple collection, low sample volume, and ambient stability, which make it particularly suited for use in remote or resource-limited settings. While ELISA-FXIIa also showed strong performance, its broader clinical adoption is constrained by limited availability and standardization.

In clinical practice, DBS may serve as a valuable frontline tool for the biochemical confirmation of HAE-C1INH, complementing existing protocols. For patients with HAE-nC1INH, genetic testing remains essential for diagnosis. Future research should prioritize the validation of these functional assays in larger, diverse populations and explore the integration of emerging biomarkers to further improve diagnostic accuracy across all HAE subtypes.

## Data Availability

The datasets presented in this study can be found in online repositories. The names of the repository/repositories and accession number(s) can be found in the article/**Supplementary Material.**
