# Supplementary material for: Evaluating functional C1INH with multiple laboratory methods across Hereditary Angioedema types
Source: Front Immunol. 2025 Aug 26;16:1654078. doi: 10.3389/fimmu.2025.1654078 (PMC12417112; doi:10.3389/fimmu.2025.1654078)
Supplement: Supplementary file 1 [file Table1.docx]

| **Initials**  **(Unaffected related)** | **Age(y)** | **Sex** | **fC1INH% Chromogenic**  **(nl ≥50%)** | **fC1INH % DBS**  **(nl ≥50%)** | **fC1INH % Pka**  **(nl ≥50%)** | **fC1INH% FXIIa**  **(nl ≥50%)** |
| --- | --- | --- | --- | --- | --- | --- |
| 1. RPF | 23 | M | 120.00 | 52.7 | 61 | NA |
| 1. PHSF | 26 | M | 121.20 | 134.78 | 387 | 109 |
| 1. MESF | 11 | F | 79 | 82.29 | 73 | 86 |
| 1. PSA | 86 | F | 132.90 | 53.4 | 152 | 70 |
| 1. RCN | 52 | F | 117.00 | 93.4159 | NA | 92 |
| 1. MPC | 9 | F | 107.00 | 114.3 | 168 | 55 |
| 1. SGA | 3 | M | 120.00 | 135.7 | 341 | 102 |
| 1. EGK | 17 | M | 119.60 | 108.9 | 341 | NA |
| 1. GSSS | 22 | M | 120.60 | 112.31 | 413 | NA |
| 1. LBM | 10 | M | 67.20 | 87.19 | NA | NA |
| 1. MB | 5 | F | NA | 98.3 | 132 | 65 |
| 1. EMJ | 79 | M | 93.90 | 69.5 | 89 | NA |
| 1. GAS | 40 | M | 120.00 | 74.6 | 91 | 62 |
| 1. AMN | 14 | M | 120.00 | 91.57 | 120 | 61 |
| 1. CRSS | 27 | F | 127.90 | 106.26 | 135 | NA |

NA: Not available

| **Initials**  **(Population control)** | **Age(y)** | **Sex** | **fC1INH % DBS**  **(nl ≥50%)** | **fC1INH % Pka**  **(nl ≥50%)** | **fC1INH% FXIIa**  **(nl ≥50%)** |
| --- | --- | --- | --- | --- | --- |
| 1. CGBL | 37 | F | 113.59 | NA | 116 |
| 1. ASO | 36 | F | 93.92 | 81 | 135 |
| 1. VMRG | 49 | F | 125.40 | 85 | 128 |
| 1. BOV | 34 | F | 72.88 | 50 | 209 |
| 1. MLOS | 68 | F | 125.27 | 86 | 112 |
| 1. ANC | 54 | F | 102.74 | 80 | 80 |
| 1. MTAR | 75 | F | 102.42 | 75 | 73 |
| 1. DQR | 25 | F | 83.52 | 79 | 92 |
| 1. GMS | 27 | F | 72.88 | 71 | 102 |
| 1. GSLZ | 35 | M | 102.42 | 95 | 95 |
| 1. ENSS | 40 | F | 69.64 | 52 | NA |
| 1. MFG | 52 | F | 81.25 | NA | NA |
| 1. MLDB | 33 | F | NA | 58 | 95 |
| 1. MTH | 32 | F | NA | 83 | 98 |
| 1. BPNR | 34 | F | NA | 74 | 79 |
| 1. LLR | 31 | F | NA | 73 | 132 |
| 1. QPCB | 41 | F | NA | 80 | 155 |
| 1. ELS | 36 | F | NA | 80 | 147 |
| 1. LM | 42 | M | NA | 89 | 165 |
| 1. GML | 28 | M | NA | NA | 182 |
| 1. LCG | 22 | F | NA | 73 | 74 |
| 1. KMA | 33 | F | NA | 81 | 87 |
| 1. MCSA | 22 | F | NA | 75 | 155 |
| 1. DA | 49 | F | NA | 86 | 69 |
| 1. SMB | 46 | F | NA | 78 | 121 |
| 1. JOA | 36 | F | NA | 82 | 162 |
| 1. DS | 60 | F | NA | 80 | 145 |
| 1. MFG | 51 | F | NA | 73 | 166 |
| 1. DKZ | 32 | M | NA | 88 | 164 |
| 1. KCB | 15 | F | NA | 84 | 151 |
| 1. LVAP | 20 | F | NA | 84 | 50 |
| 1. MFSS | 51 | F | NA | 71 | 69 |
| 1. WLO | 24 | M | NA | 76 | 117 |
| 1. GAS | 38 | F | NA | 80 | 123 |
| 1. ASS | 47 | F | NA | 79 | 134 |
| 1. VLBP | 52 | F | NA | 78 | 114 |
| 1. GNC | 23 | M | NA | 79 | 124 |
| 1. RCMF | 28 | M | NA | 82 | 130 |
| 1. TSO | 24 | F | NA | 59 | 104 |
| 1. DEP | 23 | F | NA | 62 | 117 |
| 1. MLSF | 60 | F | NA | 83 | 118 |
| 1. HSR | 29 | M | NA | 76 | 131 |
| 1. RSB | 35 | M | NA | 83 | 128 |
| 1. PSSR | 36 | F | NA | 82 | 118 |
| 1. LTP | 42 | F | NA | 72 | 68 |
| 1. NSB | 22 | F | NA | 72 | 76 |
| 1. KRS | 8 | M | NA | 80 | 66 |
| 1. JAF | 42 | F | NA | 83 | 74 |
| 1. FMS | 54 | F | NA | 80 | 131 |
| 1. DFMS | 48 | F | NA | 92 | 51 |
| 1. DFV | 26 | F | NA | 80 | 57 |
| 1. GML |  | F | NA | 74 | NA |
| 1. DCVP |  | F | NA | 84 | NA |
| 1. FACS |  | M | NA | 88 | NA |
| 1. GAS | 34 | F | NA | NA | 81 |
| 1. MVA | 49 | F | NA | NA | 82 |
| 1. MMBD | 70 | F | NA | NA | 81 |

NA: Not available

**Unaffected related controls Population control Final control group**

fC1INH % Chromogenic (n=14) ------------------------ fC1INH % Chromogenic (n=14)

fC1INH % DBS (n= 15) fC1INH % DBS (n= 12) fC1INH % DBS (n= 27)

fC1INH% Pka (n=13) fC1INH % Pka (n=51) fC1INH % Pka (n=64)

fC1INH% FXIIa (n=9) fC1INH% FXIIa (n=52) fC1INH% FXIIa (n=61)
