# Supplementary material for: Evaluating functional C1INH with multiple laboratory methods across Hereditary Angioedema types
Source: Front Immunol. 2025 Aug 26;16:1654078. doi: 10.3389/fimmu.2025.1654078 (PMC12417112; doi:10.3389/fimmu.2025.1654078)
Supplement: Supplementary file 2 [file Table2.docx]

| **Family** | **Patient** | **Age(y)** | **Sex** | **C4**  **mg/dl**  **(nl20-40)** | **C1INHq**  **mg/dL**  **(nl 19.5-34.5)** | **fC1INH**  **Chromogenic**  **(nl ≥50%)** | **fC1INH**  **DBS**  **(nl ≥50%)** | **fC1INH**  **Pka**  **(nl ≥50%)** | **fC1INH**  **FXIIa**  **(nl ≥50%)** | **Onset of symptoms**  **(y)** | **Location of edemas** | **Pre-treatment attack frequency** | **Duration**  **of episodes**  **(days)** | **Gravity** | **Treatment** | **Family history of HAE** | **Genetic Variant** |
| --- | --- | --- | --- | --- | --- | --- | --- | --- | --- | --- | --- | --- | --- | --- | --- | --- | --- |
| Family 1 |  | 60 | F | 6.4 | 8.9 | 48 | 11.7 | 39 | 5 | 20 | Fa,A,G | ≥1×/month | 3-5 | S | Oxandrolone 3mg/d | Y | *SERPING1* |
| Family 1 |  | 39 | F | 6.4 | 8.9 | 42.9 | 0 | 28 | 21 | 11 | A,Fa,E,G | ≥1×/month | 3-5 | S | *Berinert | Y | *SERPING1* |
| Family 1 |  | 33 | F | 6.4 | 3.8 | 40.9 | 0 | 18 | 3 | 10 | A,Fa,E,G | ≥1×/month | 1-3 | S | *Icatibant | Y | *SERPING1* |
| Family 1 |  | 34 | M | 6.4 | 5.3 | 35.2 | 5.7 | 25 | 6 | 10 | A,Fa,E,G | ≥1×/month | 3-5 | S | Tranexamic Acid 1g/d | Y | *SERPING1* |
| Family 2 |  | 21 | F | 6.4 | 5.9 | 50 | 0 | 26 | 3 | 7 | E,G | ≥1×/month | 1-3 | Mo | Tranexamic Acid 1g/d | Y | *SERPING1* |
| Family 2 |  | 46 | F | 6.4 | 10.2 | 10 | 0 | 36 | 17 | 25 | A,E | <6×/year | 1-3 | Mi | Tranexamic Acid 0.25g/d | Y | *SERPING1* |
| Family 3 |  | 44 | F | 6.4 | 6.5 | 30 | 0 | 25 | 8 | 20 | Fa,L,E | <6×/year | 1-3 | Mo | *Icatibant | Y | *SERPING1* |
| Family 3 |  | 49 | F | 6.4 | 8.2 | 43 | 0 | 44 | 20 | 19 | A,Fa,E | 6–11/year | 3-5 | Mo | Oxandrolone 3mg/d | Y | *SERPING1* |
| Family 3 |  | 44 | F | 6.4 | 3.8 | 39.8 | 0 | 25 | 9 | 23 | A,E | <6×/year | 1-3 | Mi | Oxandrolone 2.5mg/d | Y | *SERPING1* |
| Family 3 |  | 47 | F | 6.4 | 5.9 | 35 | 0 | 32 | 14 | 5 | A,E | <6×/year | 1-3 | Mo | Oxandrolone 1.5mg/d | Y | *SERPING1* |
| Family 3 |  | 77 | F | 6.4 | 7.6 | 40 | 0 | 28 | 15 | 6 | E,Fa,L | <6×/year | 3-5 | Mo | *Icatibant | Y | *SERPING1* |
| Family 3 |  | 16 | F | 10.1 | 12.8 | 11 | 0 | 36 | 18 | 6 | E,A | ≥1×/month | 3-5 | Mo | Tranexamic Acid 0.5g/d | Y | *SERPING1* |
| Family 3 |  | 7 | M | 11 | 9.5 | 59 | 0 | 21 | 17 | 1 | E,A | ≥1×/month | 3-5 | Mo | Tranexamic Acid 0.5g/d | Y | *SERPING1* |
| Family 4 |  | 37 | F | 7.3 | 7 | 49 | 0 | 46 | 14 | 10 | A,Fa,E,G | ≥1×/month | 3-5 | S | Oxandrolone 3mg/d | Y | *SERPING1* |
| Family 4 | 1. ⁑ | 3 | F | 17.1 | 13.5 | 44 | 22.1 | 44 | 39 | - | APs | - | - | APs | APs | Y | *SERPING1* |
| Family 4 |  | 69 | M | 20.6 | 14.2 | 45 | 27.9 | 55 | 34 | 8 | A,Fa,E,G | ≥1×/month | 3-5 | S | Oxandrolone 1mg/d | Y | *SERPING1* |
| Family 5 |  | 51 | F | 6.4 | 3.8 | 14 | 0 | 5 | 0 | 20 | A,Fa,E,G | ≥1×/month | 3-5 | S | Oxandrolone 5mg/d | Y | *SERPING1* |
| Family 5 | 1. ⁑ | 16 | F | 6.4 | 3.8 | 40 | 0 | 4 | 0 | 6 | A,Fa,E | <6×/year | 1-3 | Mi | *Berinert | Y | *SERPING1* |
| Family 5 |  | 47 | F | 9.14 | 8.3 | 9 | 0 | 49 | 75 | 15 | A,Fa,E | ≥1×/month | 3-5 | S | Oxandrolone u.d | Y | *SERPING1* |
| Family 5 |  | 20 | F | 12 | 10.2 | 2 | 0 | 44 | 23 | - | APs | - | - | APs | *Berinert | Y | *SERPING1* |
| Family 6 |  | 55 | M | 15 | 8.3 | 50.2 | 0 | 37 | 50 | 48 | A,E,G | <6×/year | 3-5 | Mo | *Icatibant | Y | NA |
| Family 6 |  | 57 | F | 8.23 | 9.5 | 16 | 0 | 37 | 17 | 33 | A,E,G,L | ≥1×/month | 3-5 | S | *Icatibant | Y | NA |
| Family 6 |  | 28 | M | 27.9 | 10.8 | 20 | 0 | 37 | 32 | 18 | Fa,E,G | <6×/year | 1-3 | Mo | *Icatibant | Y | NA |
| Family 6 |  | 18 | F | 15 | 12.8 | 53 | 13 | 49 | 34 | 5 | E,Fa,A | <6×/year | 1-3 | Mo | *Icatibant | Y | NA |
| Family 7 |  | 36 | F | 6.44 | 14.9 | 102 | 0 | 7 | 16 | 14 | Fa,G | 6–11/year | 1-3 | Mo | Danazol u.d. | Y | NA |
| Family 7 |  | 67 | F | 11 | 8.9 | 72 | 6.7 | 25 | 10 | 38 | Fa,A,E,G,L | ≥1×/month | 1-3 | Mo | Danazol u.d. | Y | NA |
| Family 7 | 1. ⁑ | 44 | M | 18.3 | 12.8 | 47 | 78.4 | 92 | 15 | 15 | Fa,A,E,G | ≥1×/month | 3-5 | Mo | Danazol u.d. | Y | *SERPING1* |
| Family 8 |  | 40 | F | 6 | 4.8 | 7 | 0 | 20 | 17 | 8 | E,A,Fa,L | <6×/year | 3-5 | Mo | NA | Y | *SERPING1* |
| Family 8 | 1. ⁑ | 7 | M | 11 | 8.9 | 52.6 | 0 | 0 | 12 | NA | APs | - | - | APs | APs | Y | *SERPING1* |
| Family 9 |  | 51 | F | 6.4 | 6.5 | 27.5 | 0 | 32 | 3 | NA | NA | NA | NA | NA | NA | Y | *SERPING1* |
| Family 9 |  | 60 | F | 16.1 | 7.7 | 52 | 0 | 46 | 4 | 6 | E,L | <6×/year | 3-5 | Mi | Oxandrolone 2mg/d | Y | *SERPING1* |
| Family 10 | 1. ⁑ | 40 | F | 7.3 | 7.7 | 33.3 | 0 | 93 | 1 | 12 | Fa,E | ≥1×/month | 3-5 | M | Oxandrolone 2.5mg/d | Y | *SERPING1* |
| Family 10 | 1. ⁑ | 20 | F | 6.4 | 11.5 | 26 | 0 | 21 | 3 | 1 | A,E | <6×/year | 1-3 | Mi | NA | Y | *SERPING1* |
| Family 10 |  | 49 | F | 12 | 7 | 49 | 0 | 41 | 17 | 15 | A,Fa,E,G | ≥1×/month | 3-5 | S | *Icatibant | Y | *SERPING1* |
| Family 11 |  | 59 | M | 12 | 5.9 | 53.6 | 0 | 36 | 7 | 30 | A,G | <6×/year | 1-3 | Mi | Danazol 0.1g/d | Y | *SERPING* |
| Family 11 |  | 61 | F | 11 | 10.2 | 8 | 0 | 55 | 9 | 16 | Fa, E | ≥1×/month | 1-3 | S | Danazol u.d. | Y | *SERPING* |
| Family 11 |  | 10 | F | 11 | 7.7 | 52 | 0 | 31 | 3 | NA | APs | - | - | APs | APs | Y | NA |
| Family 11 |  | 16 | F | 10.1 | 14.2 | 46.5 | 0 | 28 | 6 | 7 | A,Fa,E | <6×/year | 1-3 | Mi | NA | Y | *SERPING1* |
| Family 12 | 1. ⁑ | 53 | M | 6.4 | 8.8 | 10 | 0 | 47 | 17 | 2 | E | <6×/year | 1-3 | M | * Danazol | Y | *SERPING1* |
| Family 13 |  | 58 | F | 11 | 9.5 | 73 | 0 | 41 | 15 | 14 | A,E,Fa,G | <6×/year | 1-3 | M | Oxandrolone 2mg/d | Y | *SERPING1* |
| Family 14 |  | 49 | M | 13.1 | 8.8 | 33 | 0 | 45 | 39 | 14 | A,E | <6×/year | 1-3 | Mi | Oxandrolone 2mg/d | Y | *SERPING1* |
| Family 15 |  | 47 | F | 13.1 | 10.8 | 18 | 0 | 37 | 14 | 5 | E,Fa,A | <6×/year | 1-3 | Mo | Oxandrolone 2mg/d | Y | *SERPING1* |
| Family 16 |  | 27 | M | 10.1 | 12.8 | 28 | 0 | 0 | 19 | 6 | A,E,G | ≥1×/month | 1-3 | Mo | Oxandrolone 7.5mg/d | N | *SERPING1* |
| Family 17 |  | 45 | F | 12 | 12.1 | 96 | 0 | 43 | 24 | 29 | A,E,G | <6×/year | 1-3 | Mo | Tranexamic Acid 0.5g/d | Y | *SERPING1* |
| Family 18 |  | 32 | M | 13.1 | 12.8 | 26 | 0 | 51 | 14 | 1 | E | <6×/year | 3-5 | Mi | Oxandrolone 1mg/d | N | *SERPING1* |
| Family 19 |  | 55 | F | 6.44 | 7.1 | 11 | 0 | 0 | 74 | 2 | Fa, A,G | <6×/year | 1-3 | Mo | Oxandrolone 5mg/d | Y | *SERPING1* |
| Family 20 |  | 38 | F | 13.1 | 9.5 | 66.5 | 0 | 19 | 19 | 12 | E,Fa | <6×/year | 1-3 | Mi | *Icatibant | Y | *SERPING1* |
| Family 21 |  | 50 | M | 21.7 | 6.5 | 51 | 0 | 45 | 18 | 1 | Fa,E | <6×/year | 3-5 | Mo | Danazol u.d. | N | *SERPING1* |
| Family 22 |  | 54 | F | 9.14 | 9.5 | 32.5 | 0 | 21 | 0 | 7 | A,Fa,E | <6×/year | 3-5 | Mo | Danazol 0.2g/d | N | *SERPING1* |
| Family 23 |  | 72 | F | 21.7 | 10.8 | 18 | 13.9 | 63 | 22 | 12 | A,Fa,E,G | ≥1×/month | 3-5 | Mo | Danazol 0.3g/d | Y | NA |
| Family 24 |  | 14 | F | 9.14 | 5.9 | 6 | 0 | 30 | 2 | NA | APs | - | - | APs | APs | Y | *SERPING1* |
| Family 24 | 1. ⁑ | 6 | M | 10.1 | 8.8 | 30 | 0 | 15 | 0 | NA | APs | - |  | APs | APs | Y | *SERPING1* |
| Family 24 |  | 48 | M | 15 | 7.1 | 47.6 | 0 | 0 | 3 | 15 | Fa,E | ≥1×/month | 1-3 | Mo | NA | Y | *SERPING1* |
| Family 30 |  | 10 | F | 6.4 | 8.9 | 21.8 | 0 | 48 | 3 | 9 | Fa | <6×/year | 1-3 | Mi | Tranexamic Acid u.d. | Y | NA |
| Family 25 |  | 12 | M | 6.4 | 11.5 | 58 | 0 | 46 | 14 | 1 | A | <6×/year | 3-5 | Mi | Tranexamic Acid u.d. | Y | *SERPING1* |
| Family 25 | 1. ⁑ | 34 | F | 7.31 | 13.5 | 12 | 0 | 46 | 11 | 15 | Fa,L | <6×/year | 1-3 | Mo | *Icatibant | Y | *SERPING1* |
| Family 25 |  | 39 | F | 6.4 | 7.1 | 50 | 0 | 65 | 9 | 13 | A | ≥1×/month | 3-5 | Mo | Oxandrolone 2.5mg/d | Y | *SERPING1* |
| Family 25 |  | 57 | F | 12 | 4.8 | 5 | 0 | 37 | 6 | 8 | A,Fa,E,G | 6–11/year | 1-3 | Mo | Danazol u.d. | Y | *SERPING1* |
| Family 24 |  | - | F | 12 | 8.9 | 35 | 0 | 0 | NA | 1 | Fa,E | 6–11/year | 1-3 | Mo | NA | Y | *SERPING1* |
| Family 25 | 1. ⁑ | 3 | F | 8.23 | 10.8 | NA | 0 | 94 | 20 | 1 | E | 1-3/in life | 1-3 | Mi | *Berinert | Y | *SERPING1* |
| Family 26 |  | 42 | F | 22.9 | 11.5 | NA | 12.9 | 93 | 23 | 26 | E | ≥1×/month | 3-5 | Mo | Danazol 0.2g/d | Y | NA |
| Family 27 |  | 54 | M | 10.1 | 12.1 | NA | 0 | 52 | 11 | 8 | A,Fa,E,G | 6–11/year | 3-5 | Mi | NA | Y | NA |
| Family 27 |  | 25 | M | 12 | 14.9 | NA | 0 | 43 | 17 | 10 | E,A | < 6 × /year | 1-3 | Mi | NA | Y | NA |
| Family 28 |  | 44 | F | 15 | 12.8 | NA | 8.9 | 86 | 18 | 5 | A,Fa,E,G | ≥1×/month | 3-5 | Mo | Danazol 0.1g/d | Y | NA |
| Family 28 |  | 79 | F | 11 | 8.9 | NA | 0 | 44 | 3 | 5 | A,Fa,E,G | <6×/year | 3-5 | Mo | NA | Y | NA |
| Family 29 |  | 31 | M | 12 | 9.5 | NA | 0 | 60 | 4 | 19 | A,Fa,E,G | 6–11/year | 3-5 | Mo | Danazol 0.2g/d | Y | NA |
| Family 29 |  | 58 | F | 17.1 | 10.2 | NA | 0 | 73 | 6 | 1 | A,Fa,E,G | ≥1×/month | 3-5 | S | Oxandrolone 3mg/d | Y | NA |
| Family 29 |  | 30 | M | 9.14 | 9.5 | NA | 0 | 58 | 0 | 7 | A,E | 6–11/year | 3-5 | Mo | Danazol 0.4g/d | Y | NA |
| Family 29 |  | 57 | F | 18.3 | 9.5 | NA | 0 | 81 | 9 | 19 | A,Fa,E,G | <6×/year | 3-5 | Mo | Tranexamic Acid 1g/d | Y | NA |
| Family 29 |  | 55 | F | 6.4 | 8.3 | NA | 0 | 55 | 4 | 23 | Fa,E,G | <6×/year | 3-5 | Mi | NA | Y | NA |
| Family 29 |  | 29 | M | 7.31 | 9.5 | NA | 0 | 62 | 8 | 23 | Fa | <6×/year | 3-5 | Mi | Danazol 0.1g/d | Y | NA |
| Family 30 |  | 42 | F | 18.3 | 14.9 | NA | 12.5 | 47 | 14 | 6 | Fa, E, A | ≥1×/month | 3-5 | Mo | Danazol u.d. | Y | NA |

N= 72 Patient

NA: Not available; S: Severe; Mo: Moderate; Mi: Mild; APs – Asymptomatic Patients; Y: Yes; N: No; A: Abdomen; Fa: Face; E: Extremities; G: Genitals; L: Larynx

⁑ New diagnoses; * On-demand treatment; u.d.: unknown dose.
