# Supplementary material for: Evaluating functional C1INH with multiple laboratory methods across Hereditary Angioedema types
Source: Front Immunol. 2025 Aug 26;16:1654078. doi: 10.3389/fimmu.2025.1654078 (PMC12417112; doi:10.3389/fimmu.2025.1654078)
Supplement: Supplementary file 3 [file Table3.docx]

| **Family** | **Patient** | **Age(y)** | **Sex** | **C4**  **mg/dl**  **(nl20-40)** | **C1INHq**  **mg/dL**  **(nl 19.5-34.5)** | **fC1INH**  **Chromogenic**  **(nl ≥50%)** | **fC1INH**  **DBS**  **(nl ≥50%)** | **fC1INH**  **Pka**  **(nl ≥50%)** | **fC1INH**  **FXIIa**  **(nl ≥50%)** | **Onset of symptoms**  **(y)** | **Location of edemas** | **Pre-treatment attack frequency** | **Duration**  **of episodes**  **(days)** | **Gravity** | **Treatment** | **Family history of HAE** | **Genetic Variant** |
| --- | --- | --- | --- | --- | --- | --- | --- | --- | --- | --- | --- | --- | --- | --- | --- | --- | --- |
| Family 31 | 1. ⁑ | 16 | F | 6.4 | 34.2 | 46 | 0 | 63 | 9 | 14 | A,Fa,E | <6×/year | 3-5 | Mi | *Icatibant | Y | *SERPING1* |
| Family 31 | 1. ⁑ | 30 | M | 10.1 | 36 | 35 | 0 | 68 | 8 | 5 | A,Fa,E | ≥1×/month | 1-3 | Mo | *Icatibant | Y | *SERPING1* |
| Family 31 | 1. ⁑ | 51 | F | 11 | 40.6 | 19 | 0 | 54 | 10 | 15 | A,Fa,E | 6–11/year | 3-5 | Mi | NA | Y | *SERPING1* |
| Family 32 | 1. # | 56 | F | 6.4 | 13.5 | 66 | 0 | 95 | 0 | 1 | E | ≥ 1 × /month | 3-5 | Mo | *Berinert | Y | *SERPING1* |
| Family 32 |  | 11 | M | 17.1 | 10.8 | 59 | 22.8 | 58 | 0 | 6 | E,A | 6–11/year | 1-3 | Mo | Tranexamic Acid u.d. | Y | *SERPING1* |
| Family 33 |  | 22 | F | 6.4 | 21.3 | 34.5 | 0 | 38 | 28 | 10 | A,Fa,E,G | ≥ 1 × /month | 3-5 | S | Oxandrolone 7.5mg/d | N | *SERPING1* |
| Family 34 | 1. ⁑ | 25 | F | 5 | 70 | 61 | 3.6 | 41 | 95 | 6 | E,Fa,A | < 6 × /year | 3-5 | Mo | Tranexamic Acid u.d. | Y | *SERPING1* |
| Family 35 |  | 33 | F | 11 | 24.7 | 33.4 | 0 | 38 | 4 | 1 | A,Fa,E,G,L | ≥1×/month | 3-5 | S | Lanadelumab 300mg/m | Y | *SERPING1* |
| Family 35 |  | 68 | M | 15 | 49.6 | 38 | 0 | 80 | 17 | 16 | A,Fa,E,G,L | ≥1×/month | 3-5 | S | Oxandrolone 5mg/d | Y | *SERPING1* |
| Family 35 |  | 30 | M | 11 | 39.5 | NA | 0 | 57 | 6 | 9 | A,Fa,E | <6×/year | 3-5 | Mo | Oxandrolone 2.5mg/d | Y | *SERPING1* |
| Family 35 | 1. ⁑ | 40 | M | 10.1 | 29.4 | NA | 0 | 53 | 5 | 5 | A,Fa,E | <6×/year | 3-5 | Mi | Oxandrolone 2.5mg/d | Y | *SERPING1* |
| Family 36 |  | 44 | F | 7.3 | 25.6 | NA | 0 | 143 | 8 | 5 | Fa | <6×/year | 3-5 | Mi | Tranexamic Acid u.d. | Y | NA |

**N= 12 Patient**

NA: Not available; S: Severe; Mo: Moderate; Mi: Mild; Y: Yes; N: No; A: Abdomen; Fa: Face; E: Extremities; G: Genitals; L: Larynx

⁑ New diagnoses; * On-demand treatment; u.d.: unknown dose. # Patient experienced an angioedema attack during sample collection
