# Supplementary material for: Evaluating functional C1INH with multiple laboratory methods across Hereditary Angioedema types
Source: Front Immunol. 2025 Aug 26;16:1654078. doi: 10.3389/fimmu.2025.1654078 (PMC12417112; doi:10.3389/fimmu.2025.1654078)
Supplement: Supplementary file 4 [file Table4.docx]

| **Family** | **Patient** | **Age(y)** | **Sex** | **C4**  **mg/dl**  **(nl20-40)** | **C1INHq**  **mg/dL**  **(nl 19.5-34.5)** | **fC1INH**  **Chromogenic**  **(nl ≥50%)** | **fC1INH**  **DBS**  **(nl ≥50%)** | **fC1INH**  **Pka**  **(nl ≥50%)** | **fC1INH**  **FXIIa**  **(nl ≥50%)** | **Onset of symptoms**  **(y)** | **Location of edemas** | **Pre-treatment attack frequency** | **Duration**  **of episodes**  **(days)** | **Gravity** | **Treatment** | **Family history of HAE** | **Genetic Variant** |
| --- | --- | --- | --- | --- | --- | --- | --- | --- | --- | --- | --- | --- | --- | --- | --- | --- | --- |
| Family 37 |  | 62 | F | 22.9 | 21.3 | 120 | 84.8 | 74 | 74 | 20 | Fa, A | <6×/year | 1-3 | Mod | *Icatibant | Y | *FXII* |
| Family 37 |  | 70 | F | 27.9 | 43.9 | 121 | 81.5 | 154 | 42 | 32 | Fa | <6×/year | 1-3 | Mi | *Icatibant | Y | *FXII* |
| Family 37 |  | 47 | M | 26.6 | 42.8 | 147 | 73.8 | 151 | 44 | APs | APs | APs | APs | APs | APs | Y | *FXII* |
| Family 37 |  | 40 | M | 21.7 | 35.2 | 111 | 84.8 | 136 | 42 | 1 | A, E, Fa | 6–11/year | 1-3 | Mi | Tranexamic Acid 0.25g/d | Y | *FXII* |
| Family 37 |  | 48 | M | 34.6 | 38.3 | 131 | 36.4 | 141 | 34 | APs | APs | APs | APs | APs | APs | Y | *FXII* |
| Family 37 |  | 34 | F | 33.2 | 27.4 | 99 | 59.1 | 157 | 36 | 23 | Fa | <6×/year | 1-3 | Mi | NA | Y | *FXII* |
| Family 37 |  | 27 | F | 43.3 | 34.2 | 130 | 78 | 145 | 54 | 2 | Fa, E, A, G | <6×/year | 1-3 | Mo | *Icatibant | Y | *FXII* |
| Family 37 |  | 18 | M | 34.6 | 39.5 | 117 | 85 | 143 | 59 | APs | APs | APs | APs | APs | APs | Y | *FXII* |
| Family 37 |  | 57 | M | 41.8 | 40.6 | 154 | 94 | 156 | 68 | APs | APs | APs | APs | APs | APs | Y | *FXII* |
| Family 37 |  | 55 | F | 46.5 | 39.5 | 135 | 104 | 166 | 71 | NA | NA | NA | NA | NA | NA | Y | *FXII* |
| Family 37 |  | 64 | F | 48 | 29.4 | 71 | 101 | 169 | 72 | NA | NA | NA | NA | NA | NA | Y | *FXII* |
| Family 37 |  | 38 | F | 40.4 | 29.4 | 138 | 105 | 131 | 65 | 28 | A, E, Fa | 6–11/year | 1-3 | Mi | APs | Y | *FXII* |
| Family 37 |  | 40 | F | 33.2 | 29.4 | 90 | 37 | 0 | 15 | 33 | A,E | <6×/year | 3-5 | Mo | *Berinert/Pregnant | Y | *FXII* |
| Family 38 |  | 31 | F | 33.2 | 29.4 | 80.4 | 19.3 | 0 | 23 | 23 | F, E, G | <6×/year | 1-3 | Mi | *Berinert/Pregnant | Y | *FXII* |
| Family 39 |  | 34 | F | 21.7 | 34.2 | 131 | 0 | 158 | 71 | 2 | Fa, A | <6×/year | 1-3 | Mi | *Berinert/Pregnant | Y | *FXII* |
| Family 40 | 1. ⁑ | 30 | F | 38 | 40.6 | 111 | 75 | 149 | 58 | NA | APs | APs | APs | APs | APs | Y | *FXII* |
| Family 40 |  | 52 | F | 24.2 | 36.3 | 126 | 56 | 122 | 55 | 24 | A, E, Fa, L | <6×/year | < 6 × /year | Mo | NA | Y | *FXII* |
| Family 41 |  | 61 | M | 40.4 | 40.6 | 151 | 65 | 170 | 63 | APs | APs | APs | APs | APs | APs | Y | *FXII* |
| Family 41 |  | 33 | F | 30.6 | 18.9 | 103 | 83 | 73 | 50 | 21 | A, Fa, E | 6–11/year | 3-5 | Mo | Tranexamic Acid 0.5g/d | Y | *FXII* |
| Family 41 | 1. ⁑ | 6 | M | 27.9 | 45 | 60.40 | 126 | 402 | 72 | 4 | A | <6×/year | 1-3 | Mi | NA | Y | *FXII* |
| Family 41 | 1. ⁑ | 10 | M | 33.2 | 34.2 | 124 | 115 | 330 | 62 | 6 | A | <6×/year | 1-3 | Mi | NA | Y | *FXII* |
| Family 42 | 1. ⁑ | 36 | F | 36 | 33.2 | 131 | 68.3 | 148 | 44 | 14 | Fa, A | 6–11/year | 3-5 | Mi | Tranexamic Acid 0.5g/d | Y | *FXII* |
| Family 42 | 1. ⁑ | 73 | F | 44.9 | 16.4 | 120 | 94.7 | 69 | 62 | APs | APs | APs | APs | APs | APs | Y | *FXII* |
| Family 43 |  | 66 | F | 32 | 34.5 | 136 | 72.2 | 180 | 60 | 30 | A, E, Fa, L | ≥1×/month | 3-5 | Mo | Danazol | Y | *FXII* |
| Family 43 |  | 36 | F | 27.9 | 28.4 | 132.70 | 114.1 | 128 | 56 | 12 | A, E, Fa, L | ≥1×/month | 3-5 | Mo | Oxandrolone 7.5mg/d | Y | *FXII* |
| Family 44 |  | 40 | F | 40.4 | 29.4 | 152 | 82.1 | 161 | 47 | 20 | A, E | <6×/year | 1-3 | Mi | NA | Y | *FXII* |
| Family 44 |  | 37 | F | 34.6 | 28.4 | 120 | 58.5 | 134 | 56 | 18 | A, E | <6×/year | 1-3 | Mi | NA | Y | *FXII* |
| Family 46 |  | 28 | F | 36 | 37. 3 | 96.50 | 43.6 | 138 | 65 | 16 | E, Fa | 6–11/year | 1-3 | Mi | Tranexamic Acid 0.5g/d | Y | *FXII* |
| Family 47 |  | 37 | F | 22.9 | 33.2 | 131.20 | 72.7 | 128 | 66 | 15 | A, E, Fa, L | 6–11/year | 1-3 | Mo | NA | Y | *FXII* |
| Family 48 |  | 43 | F | 41.8 | 34.2 | 120 | 89.74 | 78 | 66 | 20 | Fa, E | <6×/year | 1-3 | Mi | NA | N | *FXII* |
| Family 50 |  | 52 | F | 40.4 | 20.5 | 121.4 | 72.3 | 115 | 9 | 15 | Fa, E, G, L | ≥1×/month | 1-3 | Mo | Oxandrolone 7.5mg/d | Y | *FXII* |
| Family 51 |  | 63 | F | 40.4 | 35.2 | 127 | 32 | 433 | 69 | 26 | A, Fa, E | 6–11/year | 3-5 | Mo | Tranexamic Acid 1g/d | Y | *FXII* |
| Family 51 |  | 37 | F | 26.6 | 16.4 | 87 | 74 | 354 | 66 | 19 | A, E | 6–11/year | 1-3 |  | Tranexamic Acid 0.5g/d | Y | *FXII* |
| Family 51 | 1. ⁑ | 40 | M | 34.6 | 36.3 | 122 | 112 | 381 | 85 | 25 | A | <6×/year | 1-3 | Mi | NA | Y | *FXII* |
| Family 51 | 1. ⁑ | 37 | F | 17.1 | 21.3 | 79 | 65.4 | 134 | 33 | 15 | Fa, L | <6×/year | 1-3 | Mo | *Icatibant | Y | *FXII* |
| Family 51 | 1. ⁑ | 18 | M | 21.7 | 24.7 | 84 | 66.5 | 62 | 12 | APs | APs | APs | APs | APs | APs | Y | *FXII* |
| Family 51 | 1. ⁑ | 53 | M | 22.9 | 29.4 | 81 | 44.9 | 57 | 16 | APs | APs | APs | APs | APs | APs | Y | *FXII* |
| Family 51 | 1. ⁑ | 20 | F | 18.1 | 23 | 69 | 67.4 | 64 | 4 | 18 | A, E | <6×/year | 1-3 | Mi | NA | Y | *FXII* |
| Family 53 |  | 39 | F | 34.6 | 16.4 | 109.2 | 56.82 | 108 | 55 | 14 | A,Fa,E,G,L | ≥1×/month | 3-5 | Mo | Tranexamic Acid 0.5g/d | Y | *FXII* |
| Family 53 |  | 51 | F | 25.4 | 27.4 | 119.5 | 24.78 | 82 | 26 | 25 | Fa, E, G, L | <6×/year | 3-5 | Mo | NA | Y | *FXII* |
| Family 53 | 1. ⁑ | 20 | M | 15 | 26.5 | 122.8 | 93.39 | 90 | 34 | APs | APs | APs | APs | APs | APs | Y | *FXII* |
| Family 54 | 1. ⁑ | 28 | F | 27.9 | 28.4 | 120 | 39.93 | 97 | 49 | Adolescent | A, E, Fa | <6×/year | 1-3 | Mi | NA | Y | *FXII* |
| Family 56 |  | 40 | F | 29.2 | 34.2 | 100 | 94.1 | 163 | 48 | 15 | A, E | <6×/year | 1-3 | Mi | NA | Y | *FXII* |
| Family 56 |  | 76 | F | 36 | 28.4 | 85 | 103.8 | 132 | 49 | 29 | Fa, E | <6×/year | 1-3 | Mi | NA | Y | *FXII* |
| Family 51 |  | 34 | F | 22.9 | 33.2 | 108 | NA | 98 | 55 | 32 | A, Fa, E | <6×/year | 3-5 | Mi | NA | Y | *FXII* |
| Family 52 |  | 63 | F | 26.6 | 27.4 | 114.9 | NA | 116 | 59 | APs | APs | APs | APs | APs | APs | Y | *FXII* |
| Family 53 |  | 68 | F | 29.2 | 45 | 120 | NA | 88 | 68 | 52 | A, E, Fa, L | ≥1×/month | 3-5 | Mo | Tranexamic Acid 0.5g/d | Y | *FXII* |
| Family 54 | 1. ⁑ | 38 | M | 61.4 | 45 | 120 | NA | 103 | 66 | NA | Fa, L | <6×/year | 1-3 | Mi | NA | Y | *FXII* |
| Family 54 | 1. ⁑ | 22 | F | 34.6 | 36.3 | 120 | NA | 91 | 51 | NA | Fa | <6×/year | 1-3 | Mi | NA | Y | *FXII* |
| Family 54 | 1. ⁑ | 31 | F | 34.6 | 32.2 | 120 | NA | 101 | 52 | Adolescent | FA | <6×/year | 1-3 | Mi | NA | Y | *FXII* |
| Family 55 |  | 18 | M | 17.1 | 34.2 | NA | 61.3 | 130 | 66 | 16 | E | <6×/year | 1-3 | Mi | NA | Y | *FXII* |
| Family 55 |  | 27 | M | 29.2 | 39.5 | NA | 85.8 | 134 | 79 | 15 | A, Fa, E | 6–11/year | 3-5 | S | Tranexamic Acid 0.5g/d | Y | *FXII* |
| Family 56 | 1. ⁑ | 4 | M | 38.9 | 45 | 89 | 99.9 | NA | NA | APs | APs | APs | APs | APs | APs | Y | *FXII* |

N = 53 patients

NA: Not available; S: Severe; Mo: Moderate; Mi: Mild; APs – Asymptomatic Patients; Y: Yes; N: No; A: Abdomen; Fa: Face; E: Extremities; G: Genitals; L: Larynx

⁑ New diagnoses; * On-demand treatment; u.d.: unknown dose.
