# Supplementary material for: Evaluating functional C1INH with multiple laboratory methods across Hereditary Angioedema types
Source: Front Immunol. 2025 Aug 26;16:1654078. doi: 10.3389/fimmu.2025.1654078 (PMC12417112; doi:10.3389/fimmu.2025.1654078)
Supplement: Supplementary file 5 [file Table5.docx]

| **Family** | **Patient** | **Age(y)** | **Sex** | **C4**  **mg/dl**  **(nl20-40)** | **C1INHq**  **mg/dL**  **(nl 19.5-34.5)** | **fC1INH**  **Chromogenic**  **(nl ≥50%)** | **fC1INH**  **DBS**  **(nl ≥50%)** | **fC1INH**  **Pka**  **(nl ≥50%)** | **fC1INH**  **FXIIa**  **(nl ≥50%)** | **Onset of symptoms**  **(y)** | **Location of edemas** | **Pre-treatment attack frequency** | **Duration**  **of episodes**  **(days)** | **Gravity** | **Treatment** | **Family history of HAE** | **Genetic Variant** |
| --- | --- | --- | --- | --- | --- | --- | --- | --- | --- | --- | --- | --- | --- | --- | --- | --- | --- |
| Family 57 |  | 42 | F | 36 | 37.3 | 120 | 131.04 | 72 | 67 | 25 | A, Fa, E | ≥1×/month | 3-5 | Mo | Oxandrolone 5mg/d | Y | No eligible variants were detected in the exome |
| Family 57 |  | 27 | F | 22.9 | 34.2 | 120 | 74.2 | 72 | 85 | 15 | A, Fa, E,L | 6–11/year | 3-5 | Mi | *Icatibant | Y | No eligible variants were detected in the exome |
| Family 57 |  | 29 | F | 34.6 | 42.8 | 120 | 128.1 | 79 | 102 | 16 | A, Fa, E,L | 6–11/year | 3-5 | Mo | Tranexamic Acid 0.75g/d | Y | No eligible variants were detected in the exome |
| Family 57 |  | 40 | F | 40.2 | 28.4 | 120 | 100.24 | 74 | 58 | 30 | A, Fa, E | 6–11/year | 1-3 | S | Tranexamic Acid 1g/d | Y | No eligible variants were detected in the exome |
| Family 57 |  | 36 | F | 31.9 | 31.3 | 120 | 122.26 | 78 | 64 | 20 | A,E | ≥1×/month | 1-3 | Mi | *Icatibant | Y | No eligible variants were detected in the exome |
| Family 57 |  | 68 | F | 36 | 29.4 | 120 | 121.4 | 82 | 87 | 40 | A, Fa, E | 6–11/year | 1-3 | Mi | * Tranexamic Acid | Y | No eligible variants were detected in the exome |
| Family 58 |  | 56 | F | 34.6 | 23 | 120 | 62.44 | 71 | 64 | 42 | A ,Fa, E | ≥1×/month | 3-5 | Mo | *Icatibant | Y | No eligible variants were detected in the exome |
| Family 58 |  | 28 | F | 26.6 | 33.2 | 94 | 89.27 | 152 | 58 | 25 | A,E | <6×/year | 1-3 | Mi | *Icatibant | Y | No eligible variants were detected in the exome |
| Family 59 |  | 51 | F | 40.4 | 39.5 | 126 | 119.02 | 409 | 81 | 34 | A,Fa,E,L | ≥1×/month | 3-5 | Mo | Tranexamic Acid 1.5g/d | Y | VUS in ANGPT1 |
| Family 59 |  | 28 | F | 44.9 | 29.4 | 127. | 74.6 | 343 | 82 | 14 | A,F a, E | <6×/year | 1-3 | Mi | Tranexamic Acid 0.75g/d | Y | VUS in ANGPT1 |

S: Severe; Mo: Moderate; Mi: Mild; Y: Yes; N: No; A: Abdomen; Fa: Face; E: Extremities; G: Genitals; L: Larynx

* On-demand treatment
